# Porphyrin-Functionalized Zinc Oxide Nanostructures for Sensor Applications

**DOI:** 10.3390/s18072279

**Published:** 2018-07-14

**Authors:** Mohammad Ekrami, Gabriele Magna, Zahra Emam-djomeh, Mohammad Saeed Yarmand, Roberto Paolesse, Corrado Di Natale

**Affiliations:** 1 Department of Food Science, Technology and Engineering, Agricultural Campus of the University of Tehran, P. O. Box 4111, 31587-11167 Karaj, Iran; ekrami.ut@gmail.com (M.E.); emamj@ut.ac.ir (Z.E.-d.); myarmand@ut.ac.ir (M.S.Y.); 2 Department of Electronic Engineering, University of Rome Tor Vergata, Via del Politecnico 1, 00133 Roma, Italy; magna.gabriele@gmail.com; 3 Department of Chemical Science and Technology, University of Rome Tor Vergata, Via della Ricerca Scientifica, 00133 Roma, Italy; roberto.paolesse@uniroma2.it

**Keywords:** Zinc oxide nanostructure, porphyrins, chemical sensors

## Abstract

Hybrid materials made of wide band gap semiconductors and dye molecules are largely studied mainly for photovoltaic applications. However, these materials also show interesting chemical sensitivity. Zinc oxides (ZnO) and porphyrins are good examples of a metal oxide semiconductor and a dye molecule that give rise to a hybrid material with such interesting properties. ZnO has been studied for sensors, optoelectronics, electronic devices, photo-anodes for dye-sensitized solar cells, and for mechanical energy harvesting. Porphyrins, on the other side, can be synthesized in order to mimic their roles in living systems such as oxygen transport and charge transfer for catalytic processes in animals and photosynthesis in plants. This paper provides a review of the chemical sensing properties of porphyrin-capped ZnO nanostructures. The methodologies to functionalize the ZnO surface with porphyrins are illustrated with emphasis on the relationships between the material preparation and its sensing properties. The development of sensors is described through the application of the hybrid materials to different transducers.

## 1. Introduction

The recognition, quantification, and monitoring of chemical compounds in different environments have always been a major concern since they are important issues in fields ranging from environmental control to clinical diagnosis. The evolution of analytical laboratory instruments enables the detection and the quantification of target analytes with high sensitivity and resolution. These features make these instruments irreplaceable for the accurate determination of target compounds, especially when the compounds occur in complex matrices. Analytical instrumentations, however, suffer from some defects that are an obstacle for their wide exploitation: the instruments are generally costly, need experienced operators, and cannot be used in the real-field with a continuous monitoring of the target matrix. Hence, the development of simple devices that can satisfy these important requirements is becoming more and more urgent. 

Semiconducting metal oxide nanostructures have been the dominant solid-state gas sensor technology for personal, commercial, and industrial applications. They form a platform for chemiresistive gas sensors with benefits in terms of low cost, easy fabrication, and congruity with microelectronic processes [1]. The advent of nanotechnology offered a further method to improve the sensing properties of these materials. These studies gave rise to different morphologies, such as nanowire, nanorod, and nanosheet, that have been demonstrated with a wide variety of oxides [2].

As a semiconductor, ZnO has received much attention ever since its discovery in 1844. ZnO is a wide-band-gap (3.37 eV) semiconductor characterized by a large exciton binding energy (60 meV). Hexagonal wurtzite and cubic zincblende are the most typical crystalline forms [3]. 

In the past decades, ZnO has been used in many fields such as the rubber industry (fillers, activator of rubber compounds) [4], pharmaceutical and cosmetic industries (component of dental creams/powders/pastes, absorber of ultraviolet (UV) radiation) [5], textile industry [6], photocatalysis applications [7], and in electronic devices [8].

The large variety of nanostructures enables the enhancement of surface area, as well as the chemical, physical, and electronic properties. Among its useful properties, it is worth mentioning chemical stability, electrochemical coupling coefficient, range of radiation absorption, and photostability [9]. 

ZnO provides one of the greatest assortments of structures among all known materials, and the variety of methods for ZnO production makes it possible to obtain products with particles differing in shape, size, and spatial structure. The fabrication of these structures and their application as chemical sensors has been covered by several reviews in the recent past [10,11,12]. This subject is not discussed in this review where the attention is focused on the sensing properties of porphyrin-functionalized ZnO nanostructures.

## 2. Porphyrins 

The sensitive properties of porphyrins and metalloporphyrins have been intensively studied in the past [13]. This interest arose because porphyrins exhibit intense spectral response bands and possess good chemical, photo, and thermal stability. Furthermore, the electro-optical properties of porphyrins make them suitable for photovoltaic applications [14]. 

**Figure 1:**
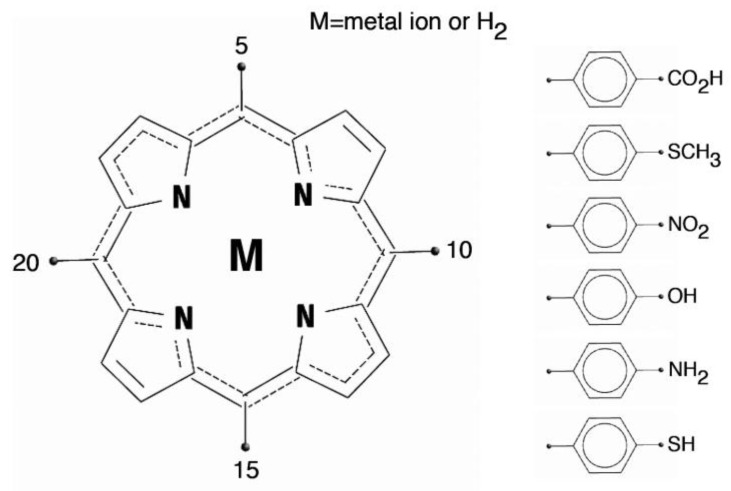
Structure of the porphyrin ring and a set of common substituents in the meso positions (5, 10, 15, 20). The central core of the porphyrin can be occupied by two hydrogen atoms (free base porphyrin) or, as in the figure, by a metal ion (metalloporphyrin).

At the synthetic level, the basic porphyrin ring is functionalized by additional groups that extend the molecular properties. The functionalization of porphyrins may involve either the core of the macrocycle or the peripheral positions. In case of the core functionalization, the four pyrroles skeleton is directly functionalized at the meso- and/or β-positions. In the other approach, functional groups are attached to the peripheral substituents. Most of these functionalizations are performed using various types of porphyrins bearing reactive substituents such as halogens, alkynes, and metals on the porphyrin core or the substituents [15]. The structure of a generic porphyrin and the porphyrin macrocycles with a set of common meso (5, 10, 15, and 20) aryl derivatives are shown in Figure 1.

Porphyrins are a versatile ligand platform, and they can interact with airborne molecules by a large variety of mechanisms. The large π-electron system allows porphyrins to act as both electron donors and acceptors [13].

Carboxylic or sulfonic groups drive the adsorption of porphyrins onto the surface of metal oxide semiconductor nanoparticles by bidentate bonding or ester-like linking. 

**Figure 2:**
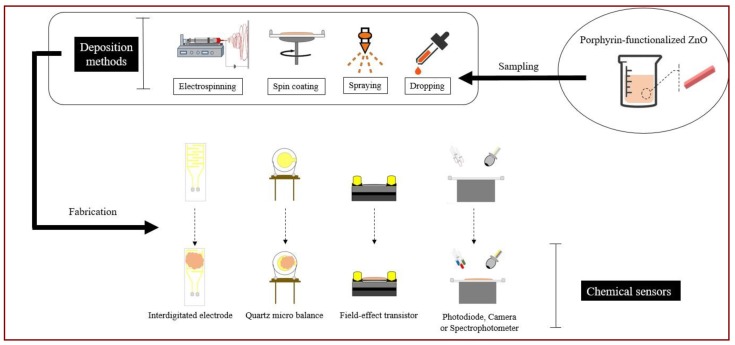
Different routes for the fabrication of devices based on porphyrin-functionalized ZnO.

Deposition techniques such as drop casting, spray, and spin coating have been made available (Figure 2). Each method has its own benefits but also drawbacks that limit the practical use to a restricted class of sensor systems.

## 3. Chemical Sensor Applications 

The typical gas sensing mechanism in ZnO involves a reaction between the airborne molecules and the ionosorbed oxygen. Surface coating with a layer of organic dyes can change this canonic usage since dye itself can activate charge transfer processes from or to the chemisorbed species. 

Pure semiconductors are virtually chemically inactive at room temperature and in dark conditions since atmospheric oxygen molecules are strongly adsorbed onto the oxide’s surface. The sensitivity in these materials is activated by high temperature or UV light irradiation. The consequent power consumption and the dependence on the stability of temperature or light intensity are considered as the main limitations of these sensors [16]. 

In n-type semiconductors the adsorption of an oxygen layer gives rise to a high resistive surface region. Along with the surface to volume ratio, the extension and modulation of this region are on the basis of the high sensitivities achieved by nanostructured materials [17].

**Figure 3:**
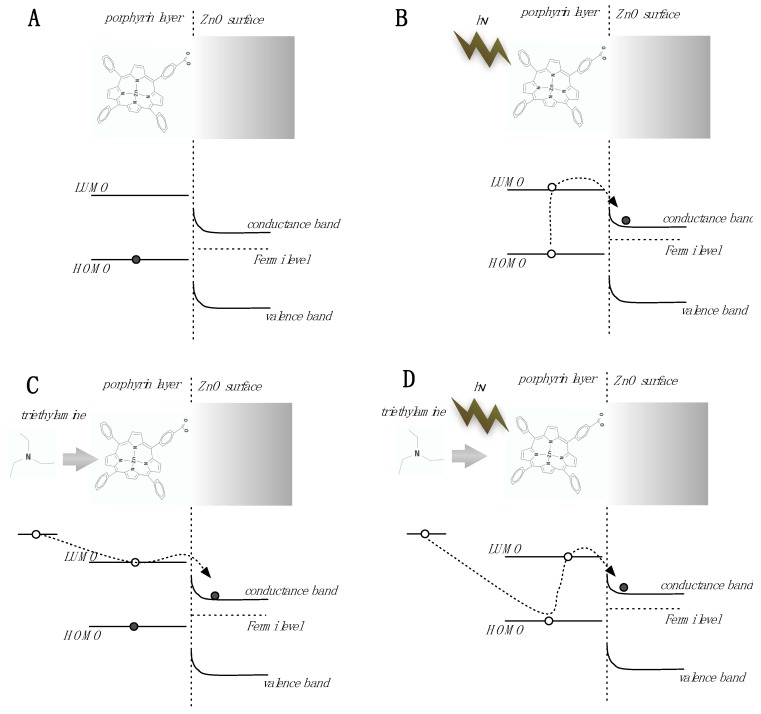
The sensitivity to light, chemicals, and the interaction between them can be understood in terms of an energy bands diagram. In dark conditions (**A**) the highest occupied molecular orbital (HOMO) level of the porphyrin lies below the Fermi level of the semiconductor, then a negligible charge transfer takes place from the porphyrin to the semiconductor. Under visible light (**B**) porphyrin electrons are excited to the lowest unoccupied molecular orbital (LUMO) level above the semiconductor Fermi level, and then the photo-excited charges can be transferred to the conduction band of the ZnO, increasing its conductivity. The adsorption in dark conditions (**C**) of an electron donor species (such as the triethylamine) results in an electron transferred to the LUMO level and then to the conductance band. Finally, under illumination (**D**) the porphyrin HOMO level is depleted by the effect of light, and then the electron transfer from the adsorpbed molecule is facilitated, since the HOMO level is deeper, and any electron at the HOMO level is photo-excited towards the conductance band of the semiconductor. The increase of electrons in the conductance band results in an increase of the conductivity and in a decrease of the surface band bending that changes the surface potential of the material. For the sake of simplicity, the changes of surface band bending are not drawn in the figures.

In the case of porphyrin-functionalization, the organic component, albeit not conductive by itself, can influence the conductivity of the underlying semiconductor [18,19]. The overall mechanism is shown in Figure 3. As known by the numerous studies on dye sensitized solar cells, porphyrins can transfer photo-electrons towards the ZnO increasing the conductivity and decreasing the surface band bending. The same mechanism can also be activated by an absorbed electron donor molecule. Under visible light, the highest occupied molecular orbital (HOMO) level of porphyrins is almost depleted of electrons and then the transfer of electrons from the absorbed molecule is expected to be more efficient. Eventually, under visible light the sensitivity increases and, most interestingly, the sensitivity towards electron donor species (e.g. amines) is enhanced. It is important to note that this effect is expected for Lewis basis. 

### 3.1. Conductometric Gas Sensors

**Figure 4:**
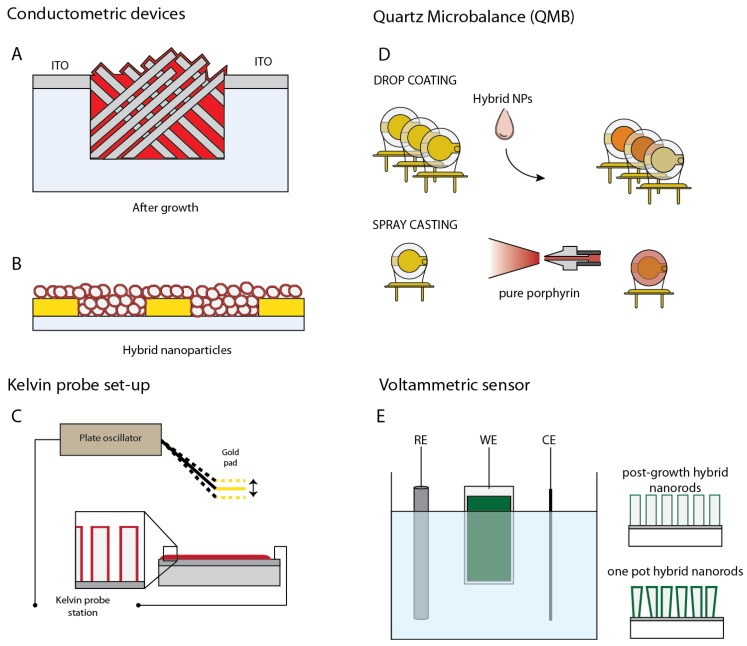
Different gas sensor configurations: (**A**) conductometric based on nanorods; (**B**) conductometric based on nanoparticles; (**C**) Kelvin probe applied to both nanorods and nanoparticles; (**D**) quartz microbalances coated with nanoparticles (NPs); (**E**) voltammetric set-up for electrodes made of hybrid co-growth and one pot functionalized nanorods.

The first approach to the sensing properties of a hybrid ZnO-porphyrin material was based on a conductometric device made of ZnO nanorods coated with a layer of Zn-porphyrins and functionalized with a carboxylic group as a linker [18]. In those device a hydrothermal grown layer of nanorods formed an intertwined forest between the indium tin oxide (ITO) electrodes gap (see Figure 4A).

Compared with pure ZnO nanorods under UV illumination, when illuminated with visible light, the functionalized materials showed a totally different behavior towards triethylamine (TEA) and ethanol. The behavior is interpreted by assuming that the porphyrins rule the sensitivity, and the adsorption of airborne molecules onto the porphyrins modulates the conductivity of the semiconductor. Remarkably, the sensitivity to triethylamine was found to be around 150 times higher than that towards ethanol. This difference is impressive considering that previous studies on a porphyrin-coated quartz microbalance and a field effect transistor reported a ratio of sensitivity to triethylamine and ethanol at 1.5 and 16.5, respectively [19]. 

Nanorods and nanowires have focused the scientific interest thanks to the possibility of fabricating mono-crystalline structures with good purity, at low-cost, and at large scale. However, the need for electrical contacts limits the implementation or applicability of these kinds of materials [20]. Hydrothermal growth requires a seed layer, by which the vertical rods are connected to the substrate. When electrodes are at the bottom, nanorods scarcely influence the overall conductivity since the electric field applied across the electrodes is mostly confined in the seed layer. The solution to this problem may require top contacts, and then a larger technological effort. Furthermore, the successive deposition of the contacts on top of the nanostructures can damage very thin nanowires and also limits the surface reachable by gases and vapors since metal layers act like a barrier to airborne molecules. 

Nanoparticles (NPs) are a viable alternative since their synthesis does not require a substrate on which to grow, as they are formed directly in solution. Unlike one-dimensional (1-D) nanostructures, NPs have very good adhesion properties so the colloidal solution can be directly dropped onto the substrate to be functionalized, such as an interdigitated electrode. After deposition, the sample is usually heated to remove lattice defects, typical of hydrothermal synthesis, and improve conductibility. 

In a recent paper [21], ZnO NPs functionalized with porphyrins are layered onto interdigitated electrodes and the change of resistance is measured after exposure to several volatile organic compounds (see Figure 4B). Four different hybrid materials were tested: they were made of four porphyrins having the same framework except for the metal coordinated to the aromatic ring. 

NPs are held together by the mutual interaction between the porphyrin layers, so in this case the response of NPs to the vapor of each compound is expected to be a combination of different interactions such as hydrogen bonds, π–π interactions, van der Waals forces, and coordination. As expected, electron donor species are a favorite, but results also show that all test compounds can be correctly discriminated by the array of sensors, even if they are presented at variable concentrations. This suggests that porphyrins-coated nanoparticles are a promising material for resistive gas sensor arrays. 

### 3.2. One-Pot Preparation of Porphyrin-ZnO Materials

**Figure 5:**
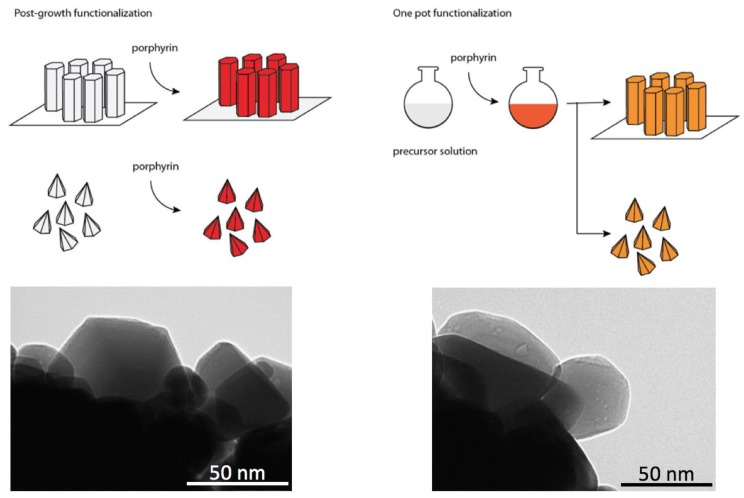
Processes of functionalization of ZnO nanorods or nanoparticles. In the post-growth process, the porphyrins are deposited from a liquid phase onto the prepared nanostructures. In the second approach, a one-pot preparation is achieved by mixing the porphyrins in the ZnO precursor solution. TEM Images show that in post-growth functionalization the hexagonal shape of the particle is preserved, while one-pot growth produces less regular inorganic structures with a more homogeneous and thinner organic layer.

Usually, the functionalization of ZnO nanostructures is achieved by immersing the ZnO in a solution containing the sensing molecules. After an appropriate period of time, ZnO is collected back and washed to remove the extra material attached to it. An alternative method for the functionalization of ZnO nanostructures consists in adding the dye directly in the precursor solution and, therefore, before the synthesis of particles (see Figure 5). Such a one-pot procedure simplifies and reduces the time required for the functionalization. Since in the co-growth procedure crystals grow under the effect of dye molecules, the resulting materials may have sensing properties that differ from those obtained with a post-growth functionalization. 

The functionalization procedure and substrate utilized for anchoring influence the overall morphology and properties of the sensing layer and, as a consequence, the sensing characteristics of the hybrid material. The first evidence of the importance of the fabrication method was obtained in the case of ZnO nanorods. In [22], the ZnO nanorods precursor solution was enriched with a water soluble porphyrin. SEM and TEM images showed that porphyrin concentration influenced the final shapes and arrangement of both nanorods and nanoparticles [23,24]. 

### 3.3. Contact Potential Difference Based Gas Sensors

The contact potential difference (CPD), also known as Volta potential, is found at the interface between two materials characterized by different Fermi levels [25]. The simplest and still useful way to measure CPD is by the Kelvin probe [26]. The Kelvin probe is a capacitor formed by the material under study and a gold plate. The CPD across the surface of the sensor and the gold plate biases the capacitor (*V_CPD_*). The gold plate is kept in a constant oscillation in order to modulate the capacitance and then to obtain a current proportional to the CPD according to the formula

$$i=\frac{dQ}{dt}=\frac{dC}{dt}V_{CPD}$$

Under the assumption that the gold plate is inert, the CPD is only influenced by the molecules adsorbed onto the sensing material. 

A Kelvin probe is typically used to measure the photovoltage, namely the change of surface potential induced by the absorption of light. This quantity in semiconductors is related to the surface band bending [27].

The effect of light on the gas adsorption properties has been studied with a Kelvin Probe set up following the evolution of the surface potential. 

In the case of porphyrin-coated ZnO, the photovoltage, namely the shift of CPD between light and dark, correlates with the electron donor properties of the adsorbed compounds.

**Figure 6:**
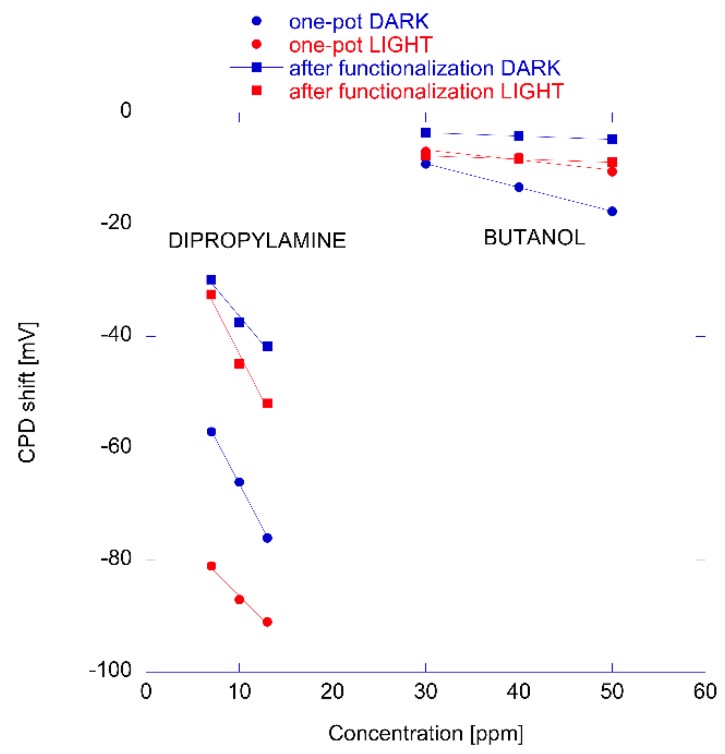
Variation of contact potential difference (CPD) in one-pot prepared material and in after-growth functionalized ZnO. The change of CPD is due to the exposure to dipropylamine and butanol and it is measured in dark and light conditions. Data from reference [22].

The Kelvin probe was further used to study the relationship between the film morphology and the sensing properties (see Figure 4C) [23]. For the scope, two sensors made of nanorods coated with porphyrins using either one pot or after growth functionalized nanorods were prepared. CPDs were measured under partial vapor pressure of 3 amines and 3 alcohols in dark and visible light conditions. Figure 6 shows the change of CPD at different concentrations of dipropylamine and butanol measured in dark and light conditions. The different behavior of the two materials can be appreciated in the plot, and in particular note that the largest difference is between light and dark shown by one-pot prepared material in the case of the electron donor compound (dipropylamine in this case). The responses to butanol are practically unchanged by the exposure to light, indicating that the sensing mechanism in this case does not involve the electron transfer but rather the CPD is due to the surface electric dipole due to the layer of adsorbed alcohol. 

Considering an array composed by the four virtual sensors (made by nanorods produced using the two growth methods and measured in visible light and dark conditions), it was found that for both materials, the difference between light and dark help to differentiate amines (strong electron donors) from alcohols whereas the porphyrin layer differences allows for the separation of the compounds within these two classes. As a consequence, the signals of the four sensors can be considered as uncorrelated, showing that changes in film morphology are sufficient to modify the sensing properties even when the same porphyrin is used.

### 3.4. Quartz Microbalance Gas Sensors

The idea to use the same porphyrin to produce different sensing materials has been further investigated with Quartz Microbalances (QMB) [24]. In this paper, 3 QMB sensors (each 20 MHz AT-cut crystals) were prepared with a porous porphyrin layer and ZnO nanoparticles functionalized with after-growth and one-pot growth methods (see Figure 4D). The same porphyrin (ZnTPPCOOH) was used in the three sensors. 

QMBs consider the whole range of interactions since mass is detected independently from the force that binds the molecule to the sensor. The array was exposed to seven different vapors of pure compounds and subsequently, as a more complex case, it was employed in an experiment aimed at detecting different cultured cells. The first evidence is that the thickness of the porphyrin layer influences the time constant of responses: thin adsorption layers such as one-pot porphyrin coatings show fast adsorption and desorption processes with respect to those of thicker layers (porphyrins and post-nanoparticle coating). In particular, the adsorption onto post growth nanoparticles shows a two steps process where the interaction with the superficial porphyrin layer is followed by the diffusion through the layer of nanoparticles. Indeed, hybrid materials can be used to improve the time response of sensors since a thin film is directly exposed to gas whereas the bulk is constituted by non-permeable material (like metal oxide crystals). Secondly, the results showed that most of the correlation between sensors is due to the concentration of the volatile compounds and the array is able to cluster the different class of compounds even with a minimal number of sensors. 

**Figure 7:**
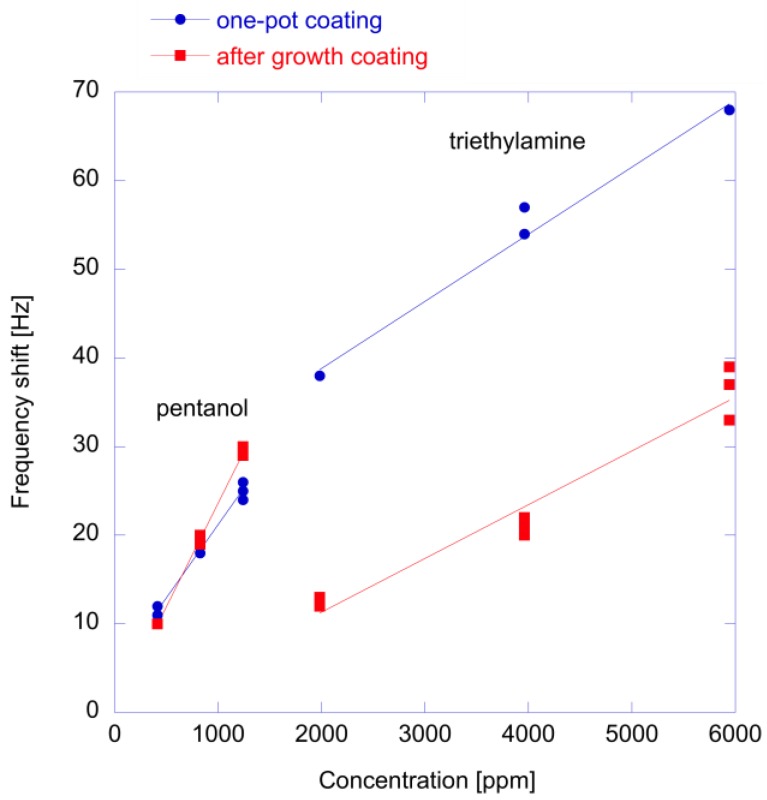
Response of QMB sensors coated with one-pot and after-growth prepared ZnTPPCOOH-functionalized ZnO nanoparticles exposed to different growing concentrations of pentanol and triethylamine. Data from reference [24].

**Figure 8:**
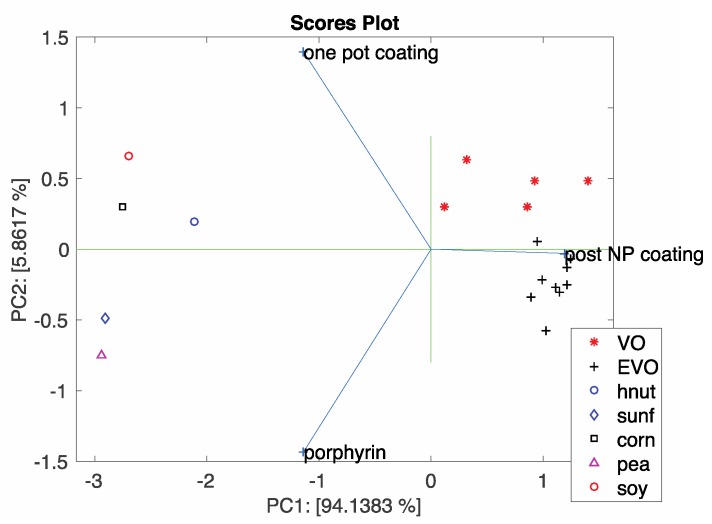
Plot of the scores and loadings of the first and second principal components of the vegetable oils experiment. Axis labels report the explained variance. Samples legend: VO: virgin olive oil; EVO: extra-virgin olive oil; hnut: hazelnut oil, sunf: sunflower oil; corn: corn oil; pea: peanut oil; soy: soybean oil.

Figure 7 shows a comparison of responses to an amine and an alcohol of the two prepared hybrid materials. Results are qualitatively similar to those obtained with the Kelvin probe and shown in Figure 8. Also in this case, the one-pot material shows a larger response to the amine while the detection of alcohols does not depend on the functionalization method. In this case, the sensitivity is not influenced by light and the measurements have always been performed in the dark. 

Optical measurements show that in one-pot prepared materials, porphyrins are less aggregated with respect to the after-growth coating [24]. This behavior is expected to favor the interaction with the metal center of the metalloporphyrin and then to increase the response to coordinating species, such as amines. 

These sensors were also used in simple electronic nose experiments aimed at discriminating complex mixture of Volatile Organic Compounds (VOCs) in a background of ambient air. For the scope, the headspace of cultures of an immortal cell line (HeLa), Mouse Embryonic Fibroblasts (MEF), and their culture medium, Dulbecco’s modified Eagle medium (DMEM), were measured. The array made of three-sensors provides a large separation between culture media and cells and a fine discrimination between these cell lines (HeLa and MEF cells). Another example of the discrimination of samples with a small array made of only three QMB sensors based on the same porphyrin but arranged on different surfaces is shown in Figure 8. The figure shows the bi-plot of the principal component analysis of data related to different vegetable oils. Analysis of loadings show the mutual information provided by the three sensors. In particular, post-coated nanoparticles are oriented towards olive oils while porphyrin-films and one-pot prepared materials are oriented towards oils of different origins.

### 3.5. Voltammetric Sensor

**Figure 9:**
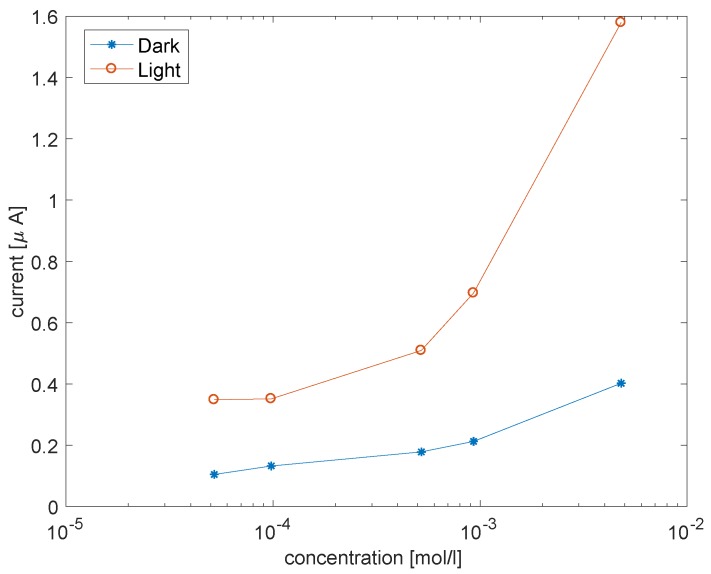
Variation of the current in a voltammetric setup as a function of the concentration of cysteine in the dark and under visible light. The voltage was kept fixed at 0.8 V. Data from reference [29].

Porphyrins as sensitive materials of electrochemical sensors have been demonstrated in several applications [13]. The combination of porphyrins and ZnO and the photoactivation of charge transfer processes is expected to activate the catalytic properties of porphyrins. These characteristics have been demonstrated in the detection of cysteine, a thiolated aminoacid important in many living processes. Porphyrins are known to favor the oxidation of thiols which results in the transfer of a charge from the sulfur atom towards the porphyrin ring. This process can be greatly enhanced in porphyrin-ZnO materials. This was demonstrated with both a free-base porphyrin on ZnO nanoparticles [28] and Mn and Cu porphyrins on nanorods [29] (set-up is shown in Figure 4E). In this last case, the exposure to visible light improves both the sensitivity and the selectivity. Figure 9 shows the current at fixed voltage as a function of the concentration of cysteine. 

[5,10,15,20-tetra(4-sulphonatophenyl)-porphyrinato] copper was used for a one-pot growth of porphyrins coated ZnO nanorods. The material was grown onto an indium tin oxide substrate that was used as a working electrode. The three-electrode amperometric system was completed by a platinum wire as counter electrode and a saturated calomel electrode (SCE, AMEL, Milan, Italy) as reference electrode.

## 4. Conclusions

The interplay between optical, electronic, and chemical properties of porphyrins and ZnO gives rise to a novel category of materials for chemical sensing. Besides the individual properties of porphyrins and ZnO, the chemical sensitivity is affected by the morphology of the material and can be controlled at the synthesis level. On the other hand, film morphology does not influence the effects of common disturbances such as temperature, which is a common mode signal that can be removed with a proper normalization. This feature is suitable for sensor array design where ensembles of sensors are operated together to capture information about complex samples. These kinds of applications are typically indicated for electronic noses or electronic tongues when sensors are operated in air or in liquid, respectively [30,31]. Brilliant examples of these applications are provided for medical diagnosis where sensor arrays are applied to breath analysis to diagnose several diseases, including some kinds of cancer [32].

Several studies demonstrated that porphyrins are versatile platforms for electronic tongue and electronic nose development [13,33]. Thus, the introduction of photo-assisted tuning for this category of sensors increases the degree of freedom for the configuration of sensor arrays. Hybrid nanostructured materials extend the sensing features of porphyrin-based sensor arrays beyond the individual constituents, pointing towards new applications, especially in the medical diagnostic field, in the control of food quality, and for signaling the presence of dangerous or toxic substances.
